# A comprehensive health-promoting neighborhood intervention to improve health care seeking behavior among reproductive age Iranian women

**DOI:** 10.1186/s12905-023-02308-0

**Published:** 2023-04-11

**Authors:** Tahereh Rahimi, Mohammad Ali Morowatisharifabad, Tahmineh Farajkhoda, Hossein Fallahzadeh

**Affiliations:** 1 Research Center for Social Determinants of Health, Jahrom University of Medical Sciences, Jahrom, Iran; 2 Elderly Health Research Center, School of Public Health, Shahid Sadoughi University of Medical Sciences, Yazd, Iran; 3 Research Center for Nursing and Midwifery Care, Shahid Sadoughi University of Medical Sciences, Yazd, Iran; 4 Center for Healthcare Data Modeling, Department of Biostatistics and Epidemiology, School of Public Health, Shahid Sadoughi University of Medical Sciences, Yazd, Iran

**Keywords:** Health care seeking behavior, Reproductive age, Women, Health promotion, Health education

## Abstract

**Background:**

Women’s health care seeking delays remains an unresolved major public health problem in developing and underdeveloped countries. This study aimed to evaluate a health-promoting neighborhood intervention to improve health care seeking behavior (HCSB) among reproductive age Iranian women using Health Promotion Model (HPM).

**Method:**

This randomized controlled trial was conducted on 160 women of reproductive age in two groups: experimental and control. Data were collected by self-administered questionnaire based on HPM constructs and a medical symptom checklist. A health-promoting neighborhood intervention was performed in seven sessions for the experimental group. HCSB and HPM constructs were measured before and 3 months after intervention in the two groups. p < 0.05 was considered significant level.

**Results:**

The average mean age of participants was 30.45 ± 7.80 years. After intervention, the mean score of self-efficacy, interpersonal influences, commitment to plan and HCSB was increased significantly in women in the experimental group, while negative constructs such as perceived barriers, negative activity-related affect, and immediate competing demands and preferences significantly decreased (p < 0.05). In addition, the mean score of referring for symptoms such as excessive sweating, persistent fatigue or weakness, headache, bleeding or spotting between periods, vaginal itching and irritation, unusual vaginal discharge, flashing, chest pain, rapid heartbeats, aching muscles or joints, urinary problems and some mental disorder was increased significantly in experimental group compared to control group (p < 0.05).

**Conclusions:**

The results of study show that an intervention based on the HPM have a positive impact on HCSB and its associated factors and can help improve women’s health behaviors and health outcomes.

## Background

Gender differences often effect on taking preventive health measures, access to health care centers, taking medicines and adherence to treatment or follow*-*up medical care [1]. However, there is evidence that show women are more likely than men to seek health care and diagnostic services, but this pattern does not occur in treating all diseases and health conditions or using specialized care services [2]. Many women around the world still do not seek timely health care, or choose informal sources of treatment for their illness or disease. The results of scientific studies show that the prevalence of delay for more than 7 days in health care seeking by women, was 39% for sexually transmitted diseases [3], 86.4% for mental disorders such as perinatal depression and emotional problem [4] and 52.9% for reproductive tract infections [5] and 70% after receiving symptoms associated with chronic illnesses [6]. Also, the rate of patient delay (length of delay ≥ 90 days) was reported 35.8% by women who noticed symptoms related to breast cancer [7].

Health care seeking behavior (HCSB) referred to activities that are performed with the aim of maintaining and promoting health, preventing illness or to deal with any deviation from good health [8]. Appropriate and on-time HCSB has tremendous potential to reduce the incidence of life-threatening diseases. Identifying the factors by which women decide why, when and where to seek treatment that may improve health programs and services [9]. Moreover, this allows medical resources to be optimally distributed, and provides better planning for access and utilization of healthcare services [10]. Despite the known importance for early interventions, delays in seeking health care and treatment remains an unresolved major public health problem in developing and underdeveloped countries. The importance of addressing this issue is that a woman’s health can be closely linked to health of her family and community [11].

There are few studies that have specifically examined the HCSB and related factors in reproductive age women, and most of them lacked theoretical framework interventions. However, a theoretical model can help the health educators in various ways, including gathering information in the stage of needs assessment, program development and create tailored content to improve the desired behavior, as well as program evaluation. A theory or model helps to identify the characteristics of the individual and the living environment that somehow affect her/his behaviors and thus increase the effectiveness of health education programs [12]. The aim of this study was to evaluate a health-promoting neighborhood intervention to improve HCSB among reproductive age Iranian women using Health Promotion Model (HPM).

### Theoretical framework

A review of the existing literature on HCSB shows that most of previous studies have no theoretical framework and only a few studies used some constructs of theories and models. For example, the role of individual factors (such as educational level, monthly income, household size) has been discussed in some studies [13, 14]. A study by Dubayova et al. described behavior-related emotions such as fear to perform HCSB [15]. The impact of social support and social media has been stated by Kim et al. [16] and Ohashi et al. [17]. Competitive barriers and demands for using formal health care services have also been noted in other studies [14, 18]. Finally, the role of situational influences or place of health care suggested by Majaj et al. [19]. According to the approach of previous studies, in this study, Pender’s Health Promotion Model (HPM) has been used as a comprehensive theoretical framework. This model includes the following constructs: personal Factors, prior related behavior, perceived benefits of action, perceived barriers of action, perceived self-efficacy, situational influences, interpersonal influences, commitment to plan of action and health promoting behavior [20].

## Methods

### Study design and participants

This randomized controlled trial was conducted on 160 women of reproductive age in Qom, Iran. Participants were selected by cluster sampling. Briefly, two municipal districts were randomly selected from eight ones, and then two health centers were randomly selected from each district so that four health centers were included in the study. Finally, 40 women of reproductive age were randomly selected from each four health centers according to inclusion criteria and randomly allocated to one control (n = 80) group and one intervention (n = 80) group. Inclusion criteria were being married, having Iranian nationality, and the age of 15–49 years (all participants were above 16 years of age) and exclusion criteria were being pregnant and having a diagnosed chronic disease or condition that need to ongoing medical therapies or long-term medical care setting.

Sample size was calculated considering the confidence level of 95%, Z_α_ = 1.96, Z_β_ = 0.84, d = 1.2 and 2.5 for standard deviation (obtained from a pilot study with 10 women who were selected to test face validity for the questionnaire). This gives a sample size of 68 subjects for each group. Finally, to increase the study power, 80 subjects were considered for each group. The formula for calculating the sample size is as follows:

$$\varvec{n}=\frac{2{\left({\varvec{z}}_{\varvec{\alpha }/2}+{\varvec{z}}_{\varvec{\beta }/2}\right)}^{2} {\varvec{\sigma }}^{2}}{{\varvec{d}}^{2}}$$

### Data collection instruments

#### 1. HPM constructs questionnaire on HCSB

To develop the HPM questionnaire, first, a qualitative study was conducted with 22 women of reproductive age and health stuff. Questionnaire items are extracted from qualitative data and also related literature. Ten women of reproductive-aged, participated in the pilot study and examined the initial scale for simplicity, clarity and readability (face validity) and then the questionnaire was modified to their opinions. Content validity rate (CVR) and content validity index (CVI) also were assessed by the expert panel with the participation of 10 people. Based on the experts’ opinions, CVI and CVR were considered acceptable with 0.94 and 0.96 respectively.

#### 1–1. Individual characteristics

this section includes age, education level, occupation, number of family members and health insurance coverage, and the way (s) of gaining health information about women’s diseases and health issues.

#### 1–2. Perceived benefits

the most important benefits of HCSB, from the perspective of women of reproductive age, include 8 items (such as *treatment in early stages is done with more convenient methods*). These items were measured on a Likert scale ranging from 1 (= completely disagree) to 5 (= completely agree).

#### 1–3. Perceived barriers

this scale includes 15 items regarding individual barriers, social barriers and barriers associated with the treatment process (such as *I don’t know the symptoms of the disease for which I should refer to a doctor*). These items were measured on a Likert scale ranging from 1 (= completely disagree) to 5 (= completely agree).

#### 1–4. Self-efficacy

this section was aimed to measure women’s judgment about their abilities to do HCSB using a 7-point scale (such as *even if no one comes with me, I can refer to the doctor alone*). These items were measured on a Likert scale ranging from 1 (= completely disagree) to 5 (= completely agree).

#### 1–5. Activity-related emotions

the emotions associated with HCSB were divided into two parts: positive emotions (3 items such as *when I refer to a doctor, my confusion is resolved and I feel safe*); and negative emotions (9 items such as *when I want to do a test or diagnostic method like MRI or endoscopy for the first time, I feel scared*). These items were measured on a Likert scale ranging from 1 (= completely disagree) to 5 (= completely agree).

#### 1–6. Interpersonal influences

according to HPM, this construct was divided into three subsections: (a) Social support (8 items such as *when I feel lazy to visit a doctor, my family members encourage me*); (b) Social norms (5 items, such as *my children expect to have a healthy mother*); and (c) Role models (3 items, such as *I learned from my mother not to treat my illnesses by myself*). These items were measured on a Likert scale ranging from 1 (= completely disagree) to 5 (= completely agree).

#### 1–7. Situational influences

to measure situational influences, 10 items were developed to investigate the location and situation in which the woman feels comfortable doing HCSB (such as *I know a health center that spends enough time to examine the patients*). These items were measured on a Likert scale ranging from 1 (= completely disagree) to 5 (= completely agree).

#### 1–8. Immediate competitive preferences and demands

Twelve items were used to measure this construct to investigate the most important preferences (such as *Instead of going to a doctor, I prefer to tolerate the disease to make my body resistant to microbes*); and immediate competitive demands (for example, *for a married woman, being present in home, and considering the needs of the spouse and children have a higher priority than visiting a doctor*) regarding HCSB in women of reproductive age. These items were measured on a Likert scale ranging from 1 (= completely disagree) to 5 (= completely agree).

#### 1–9. Commitment to plan of action

This scale consists of 7 items (such as *I intend to regularly check my physical symptoms*) to have the respondents express their level of commitment to planning for the HCSB. These items were measured on a Likert scale ranging from 1 (= completely disagree) to 5 (= completely agree).

#### 1–10. HCSB

six four-choice items were developed that addressed referral times, referral location, and treatment follow-up rate. The items were scored as 1(= never) to 4 (= always).

#### 2. Medical symptom checklist

the medical symptom checklist contains the most common signs or symptoms of disease in females. This instrument was administered to measure the mean of referring to the health care system after observing signs or symptoms during the three months. The scores were ranged between − 1(= not performing HCSB) to 1 (= performing HCSB).

### Local intervention program to improve HCSB

#### a. Development of educational materials

analysis of pre-test results revealed that many of our participants did not have information about the warning signs or symptoms of illness and the appropriate time to visit a doctor. The rate of self-treatment was high, and they only referred to the doctor if they were severely ill or felt ill for a long time; or they referred to the doctor only when vital organs were also involved. They had little information about how to manage some of the minor signs of illness at home. According to the need assessment of participants, we used several educational resources to improve their HCSB:

1. A booklet in Farsi, titled “*Guidelines for health care seeking in diseases (for women of reproductive age)”* to explain warning signs or symptoms and certain recommendations to visit the doctor or in-home disease management. This booklet was prepared using some self-care educational resources of the Ministry of Health of Iran [21].
2. Educational posters to increase knowledge and sensitivity to the intervention program installed in neighborhood health care centers and schools;
3. Educational pamphlets (for three groups of women of reproductive age, health staff and husbands):
4. Internet-based women’s health education channel containing educational text content, videos and images (also available to other family members);
5. Two native educational apps, one for the early diagnosis of the disease according to reported symptoms, and the other for locating health centers, laboratories and pharmacies across the city; The selection of educational resources was carried out according to the participants’ initial need assessment and also assessment of access to them. The resources were offered to experimental group at certain intervals until the completion of the intervention. The educational programs for experimental group were held at the health centers, and the participants of the two groups were not in contact with each other. Therefore, women from control group did not exposed to educational resources and they only received routine education from health staff.

#### b. Educational strategies

intervention was performed according to the protocol of HPM. According to this protocol, some intervention strategies are specific to one construct, and others are applicable to modifying several constructs. It may be therefore possible to use a strategy to change a construct in one session and to reinforce another construct in other sessions. Table 1 shows some examples of strategies used in our intervention.

**Table 1:** Example of strategies used in intervention based on HPM constructs

HPM constructs	Strategies
Perceived benefits	Using group discussions about the benefits of early referral to a doctor or other health care staff at the onset of illness.
Perceived barriers	Increasing knowledge about the signs or symptoms of women’s diseases to reduce cognitive barriers; explaining how to use the health system referral system to reduce health costs; introducing inexpensive or charity health centers that offer quality services; individual counseling to solve barriers
Perceived self-efficacy	Strategies for managing stress, financial issues and timely visits to the doctor.
Activity-related affect	Inviting a local woman with treated cancer to share her positive and negative emotions that she had had during the treatment process; using positive thinking techniques to overcome the fear of medical treatment (e.g., having a positive conversation with yourself, using religious recitations, thinking about positive results); playing photos and videos to become familiar with medical diagnostic methods; sharing the experiences regarding endoscopy to reduce feelings of embarrassment, fear and anxiety; strategies for contributing to patient safety and preventing the transmission of hospital infections.
Interpersonal influences	Holding the meeting *“All together for the health of women in the neighborhood”* attended by women of the experimental group and people who had supported them (such as mother, sister or daughter) at the health center and explaining health plans by health care staff and inviting them to further contribute to improving neighborhood health; development of self-assistant health groups from neighbors; introducing healthy role models and awarding prizes to them; selecting the neighborhood health volunteers to reflect the problems and needs of women to health care staff; local religious people’s support for women’s health at religious meetings by reminding health messages; teaching the principles of establishing effective communication with the patient to health care staff; sending printed pamphlets on the ways of supporting women’s health to the participants’ husbands; informing about the mental health services for counseling and assisting in resolving psychological problems.
Situational influences	Introducing an app to identify the location of nearest health centers, pharmacies and laboratories across the city; introducing diagnostic centers and hospitals that have beautiful environment and offer quality and affordable services.
Immediate competitive preferences and demands	Showing the video of “*The woman: heart of the family”* and holding a group discussion about the video’s message; providing information about medication management; practicing the skills of reading the medication brochures contain; showing a video about the complications of self-medicated chemical and herbal drugs; using self-assessment checklists on how to consume medications at home; simulating the scenario of a serious illness development for the participant and suggesting possible choices.
Commitment to plan of action	The technique of setting and prioritizing short-term objectives; practicing the skill of measuring respiration rate and pulse rate as well as the skills of using some medical equipment in small groups; training the continuous assessment of warning signs and symptoms and early diagnosis.

#### c. Implementation of the intervention

seven sessions (6 sessions for women of reproductive age and 1 session for health care staff) were held in the experimental group. The interval between sessions was one week. During the implementation of the program, the quality of the components of the program and its implementation was reviewed using the process evaluation checklist, and if necessary, changes were made to increase the likelihood of success. In addition, the assessment, which measured the change in HCSB and HPM constructs, was performed using a researcher-made questionnaire both at pre-test and 3 months after intervention in the two groups. The response rate in both stages was 100%.

### Data analysis

To analyze the results from educational intervention, parametric statistical tests were performed after the normal distribution of data was investigated by the Komomolorov-Smirnov test. Before intervention, demographic characteristics were investigated by chi-squared test, and the HPM constructs and medical symptoms in the two groups was investigated by independent *t*-test. The difference between the mean scores of HPM constructs and medical symptoms before and after intervention in each group was assessed by Paired *t*-test. Independent *t*-test was used to investigate the difference in the mean scores of HPM constructs and medical symptoms before and after the intervention between experimental and control groups. SPSS (version 20) was used to perform data analysis, and p value < 0.05 was considered significant.

## Results

The average age of participants was 30.45 ± 7.80 years. Before the intervention program, there was no significant difference in demographic characteristics, HPM constructs, and referral rate to health care staff after observing the symptoms of illness in the two groups. Table 2 shows the demographic characteristics of the participants. The results showed that after intervention, the mean score of self-efficacy, interpersonal influences, commitment to plan, and HCSB was increased significantly in women in the experimental group, while negative constructs, such as perceived barriers, negative activity-related affect, and immediate competing demands and preferences significantly decreased (p < 0.05). Although the mean scores of perceived benefits, positive activity-related affect, and situational influences was significantly increased in the experimental group compared to those before intervention (p < 0.05), these changes were not statistically significant as compared to the control group (p > 0.05) (Table 3). Table 4 shows the average referring score to the health care system after observing signs or symptoms of illness in participants. According to results, the mean score of referring for symptoms such as excessive sweating, persistent fatigue or weakness, headache, bleeding or spotting between periods, vaginal itching and irritation, unusual vaginal discharge, flashing, chest pain, rapid heartbeats, aching muscles or joints, urinary problems and some mental disorder symptoms was increased significantly in experimental group after the implementation of the educational program when compared to control group (p < 0.05).

**Table 2:** Demographic characteristics of reproductive age Iranian women

Variable		Experimental group (n = 80)		Control group (n = 80)	
		n	%	n	%
Age categories (18–49 years)	< 20 years	6	7.5	8	10
	20–29 years	32	40	37	46.2
	30–39 years	25	31.3	27	33.75
	40–49 years	17	21.2	8	10
Education level	Illiterate	2	2.5	2	2.5
	≤ 12th grade	57	71.25	60	75
	> 12th grade	21	26.25	18	22.5
Occupation	Household duties	63	78.75	64	80
	Employee	16	20	14	17.5
	Casual laborer	1	1.2	2	2.5
Family size	2	7	8.75	15	18.7
	3	23	28.75	27	33.75
	4	37	46.25	23	28.75
	≥ 5	13	16.2	15	18.75
Health insurance coverage	Yes	65	81.2	70	87.5
	No	15	18.75	10	12.5
Health information source	Radio and TV	16	20	19	23.75
	Health staff	37	46.25	38	47.5
	Print media	7	8.75	5	6.25
	Internet	20	25	18	22.5

**Table 3:** Comparison of mean and standard deviation of HCSB and PHM constructs before and after the intervention in the experimental group

HPM constructs	Group	Before intervention M ± SD	After intervention M ± SD	p value
Perceived benefits	Experimental	30.28 ± 5.97	32.11 ± 5.19	< 0.001^†^
	Control	31.93 ± 5.86	31.32 ± 5.73	0.29
	p value	0.42	0.36	
Perceived barriers	Experimental	43.95 ± 9.21	41.77 ± 9.36	< 0.001^†^
	Control	45.45 ± 9.33	46.11 ± 9.54	0.06
	p value	0.31	0.004^*^	
Perceived self-efficacy	Experimental	28.96 ± 4.35	30.27 ± 5.08	< 0.001^†^
	Control	28.46 ± 5.22	28.13 ± 5.77	0.11
	p value	0.51	0.01^*^	
Activity-related affect (positive feelings)	Experimental	12.38 ± 2.13	12.83 ± 1.50	0.01^†^
	Control	12.52 ± 2.03	12.76 ± 1.78	0.08
	p value	0.67	0.77	
Activity-related affect (negative feelings)	Experimental	26.90 ± 6.93	25.93 ± 6.60	0.003^†^
	Control	28.15 ± 6.27	28.35 ± 6.13	0.51
	p value	0.13	0.01^*^	
Interpersonal influences	Experimental	55.92 ± 7.69	60.37 ± 8.79	< 0.001^†^
	Control	56.58 ± 8.67	56.87 ± 7.83	0.44
	p value	0.61	0.009^*^	
Situational influences	Experimental	38.13 ± 4.90	39.71 ± 5.06	< 0.001^†^
	Control	38.88 ± 4.86	38.37 ± 4.78	0.06
	p value	0.33	0.08	
Competing demands and preferences	Experimental	37.30 ± 7.58	34.51 ± 6.97	< 0.001^†^
	Control	37.46 ± 6.89	37.79 ± 7.56	0.24
	p value	0.72	0.005^*^	
Commitment to plan of action	Experimental	27.20 ± 4.78	29.02 ± 4.13	< 0.001^†^
	Control	27.17 ± 4.90	27.53 ± 4.41	0.39
	p value	0.88	0.01^*^	
HCSB	Experimental	17.23 ± 3.07	18.27 ± 2.61	< 0.001^†^
	Control	17.01 ± 3.27	17.10 ± 2.93	0.15
	p value	0.40	0.001^*^	

Mean values were significantly different from those of the control group (independent-samples t test): ^*^p < 0.05.

Mean values were significantly different from those before the intervention (paired-samples t test): ^†^p < 0.05.

**Table 4:** Comparison of mean and standard deviation of referring to the health care system after observing symptoms of illness before and after the intervention in the experimental group

Symptoms of illness	Group	Before intervention M ± SE	After intervention M ± SE	p value
Fever or chills	Experimental	0.06 ± 0.03	0.05 ± 0.03	0.56
	Control	0.01 ± 0.03	0.03 ± 0.03	0.32
	p value	0.36	0.80	
Excessive sweating	Experimental	− 0.09 ± 0.04	0.05 ± 0.04	0.01^†^
	Control	− 0.13 ± 0.04	− 0.11 ± 0.04	0.56
	p value	0.76	0.04^*^	
Persistent fatigue or weakness	Experimental	− 0.1 ± 0.05	0.15 ± 0.05	0.001^†^
	Control	− 0.05 ± 0.07	− 0.04 ± 0.06	0.57
	p value	0.17	0.01^*^	
Sleep disturbances	Experimental	− 0.04 ± 0.05	0.05 ± 0.05	0.07
	Control	− 0.11 ± 0.05	− 0.05 ± 0.04	0.02^†^
	p value	0.54	0.26	
Unexplained weight loss or gain	Experimental	0.04 ± 0.03	0.01 ± 0.03	0.1
	Control	− 0.04 ± 0.03	− 0.03 ± 0.03	0.33
	p value	0.28	0.90	
Loss of appetite	Experimental	0.01 ± 0.02	0.01 ± 0.02	0.32
	Control	0.03 ± 0.02	− 0.01 ± 0.01	0.08
	p value	0.76	0.36	
Nausea or vomiting	Experimental	0.04 ± 0.02	0.06 ± 0.03	0.31
	Control	0.04 ± 0.03	0.04 ± 0.03	0.99
	p value	0.55	0.80	
Headache	Experimental	− 0.1 ± 0.06	0.14 ± 0.06	0.007^†^
	Control	− 0.1 ± 0.07	− 0.09 ± 0.06	0.78
	p value	0.97	0.02^*^	
Dizziness	Experimental	0.06 ± 0.04	0.06 ± 0.03	0.98
	Control	0.03 ± 0.04	0.06 ± 0.03	0.18
	p value	0.80	0.81	
Bleeding or spotting between periods	Experimental	0.01 ± 0.03	0.09 ± 0.03	0.03^†^
	Control	0.03 ± 0.04	− 0.05 ± 0.04	0.06
	p value	0.95	0.01^*^	
Vaginal itching, burning, and irritation	Experimental	− 0.08 ± 0.04	0.09 ± 0.04	0.008^†^
	Control	− 0.05 ± 0.05	− 0.01 ± 0.05	0.32
	p value	0.61	0.02^*^	
Heavy bleeding	Experimental	− 0.04 ± 0.03	0.1 ± 0.03	0.01^†^
	Control	0.01 ± 0.04	0.01 ± 0.04	0.82
	p value	0.21	0.12	
Moderate or severe pelvic or abdominal pain	Experimental	0.01 ± 0.03	0.03 ± 0.02	0.56
	Control	0.03 ± 0.03	0.01 ± 0.02	0.65
	p value	0.90	0.84	
Unusual vaginal discharge of any type or color or with strong odor	Experimental	− 0.1 ± 0.03	0.05 ± 0.03	0.01^†^
	Control	− 0.08 ± 0.03	− 0.09 ± 0.03	0.57
	p value	0.87	0.03^*^	
Breasts problems	Experimental	0.01 ± 0.02	0.05 ± 0.02	0.09
	Control	0.03 ± 0.02	− 0.04 ± 0.02	0.08
	p value	0.70	0.05	
Itching skin	Experimental	− 0.09 ± 0.03	0.01 ± 0.03	0.04^†^
	Control	− 0.08 ± 0.03	− 0.04 ± 0.02	0.18
	p value	0.51	0.37	
Flashing	Experimental	− 0.01 ± 0.02	0.04 ± 0.02	0.08
	Control	− 0.05 ± 0.03	− 0.05 ± 0.03	0.99
	p value	0.50	0.04^*^	
Frequent coughing	Experimental	− 0.03 ± 0.02	0.05 ± 0.02	0.08
	Control	0.03 ± 0.02	0.01 ± 0.02	0.34
	p value	0.18	0.43	
Shortness of breath	Experimental	0.05 ± 0.02	0.04 ± 0.02	0.32
	Control	0.04 ± 0.02	0.01 ± 0.02	0.15
	p value	0.60	0.36	
Chest pain	Experimental	− 0.08 ± 0.03	0.05 ± 0.03	0.01^†^
	Control	− 0.06 ± 0.03	− 0.05 ± 0.03	0.78
	p value	0.95	0.03^*^	
Rapid or skipped heartbeats	Experimental	0.01 ± 0.04	0.08 ± 0.03	0.04^†^
	Control	− 0.04 ± 0.04	− 0.08 ± 0.04	0.18
	p value	0.66	0.03^*^	
feeling empty, sad all the time, or worthless	Experimental	− 0.13 ± 0.04	0.08 ± 0.05	0.005^†^
	Control	− 0.11 ± 0.04	− 0.09 ± 0.04	0.41
	p value	0.74	0.04^*^	
Nervousness or anxiety	Experimental	− 0.14 ± 0.04	0.09 ± 0.04	0.002^†^
	Control	− 0.11 ± 0.04	− 0.13 ± 0.04	0.33
	p value	0.88	0.009^*^	
Boredom	Experimental	− 0.16 ± 0.04	0.1 ± 0.03	0.001^†^
	Control	− 0.14 ± 0.04	− 0.13 ± 0.04	0.32
	p value	0.71	0.01^*^	
Sexual difficulties	Experimental	− 0.15 ± 0.04	0.01 ± 0.04	0.01^†^
	Control	− 0.1 ± 0.03	− 0.09 ± 0.04	0.76
	p value	0.48	0.27	
Sore throat	Experimental	0.01 ± 0.01	0.03 ± 0.02	0.3
	Control	0.01 ± 0.02	0.04 ± 0.02	0.16
	p value	0.21	0.92	
Eye problems	Experimental	− 0.03 ± 0.03	0.01 ± 0.03	0.4
	Control	− 0.03 ± 0.02	− 0.01 ± 0.02	0.33
	p value	0.44	0.54	
Dental problems	Experimental	− 0.01 ± 0.04	0.08 ± 0.04	0.05
	Control	− 0.04 ± 0.04	0.03 ± 0.04	0.13
	p value	0.92	0.57	
Aching muscles or joints	Experimental	− 0.18 ± 0.06	0.08 ± 0.07	0.001^†^
	Control	− 0.13 ± 0.06	− 0.15 ± 0.06	0.56
	p value	0.86	0.04^*^	
Urinary Problems	Experimental	− 0.09 ± 0.03	0.06 ± 0.03	0.01^†^
	Control	− 0.06 ± 0.03	− 0.01 ± 0.03	0.41
	p value	0.81	0.005^*^	
Stomach pain or discomfort	Experimental	− 0.06 ± 0.04	0.05 ± 0.04	0.02^†^
	Control	0.01 ± 0.04	0.01 ± 0.04	0.91
	p value	0.34	0.75	

Mean values were significantly different from those of the control group (independent-samples t test): ^*^p < 0.05.

Mean values were significantly different from those before the intervention (paired-samples t test): ^†^p < 0.05.

## Discussion

According to the results of this study, the mean score of perceived benefits of HCSB significantly increased in the experimental as compared to that before intervention, while this increase was not statistically significant in comparison to control group. Fair et al. [22] point out that low or high level of perceived benefits alone cannot necessarily lead to receiving primary health care or treatment and other factors such as the perceived risk of disease, have a more prominent role in women’s taking action to receive treatment. Mbachu et al. considers peer education to be an effective strategy for increasing women’s perceptions of the usefulness of timely HCSB for early diagnosis of disease, and suggests that this type of training be integrated with the training of health stuffs and be continued over time [23].

After the intervention, perceived barriers in the women in experimental group significantly decreased as compared to control group. There is some evidence that community health programs, in local communities or small religious organizations, have helped increase the reception of health services by reducing perceived barriers [24, 25]. The significance of the role of perceived barriers is that evading medical care may ultimately delay the diagnosis of disease and lead to preventable consequences or premature death. In addition, reduction of perceived barriers in participants led to a significant increase in their self-efficacy. This means that women in experimental group believed they were more capable of choosing appropriate treatment and avoiding self-treatment, which is similar to the outcomes of the educational intervention in the study of Tawfik on middle-aged Egyptian women’s referral to doctor for diagnosis of type 2 diabetes [26]. Research has shown that to increase self-efficacy, behavioral activities (such as goal setting, contracting to conduct behavior and feedback from others) are more efficient than traditional practices (such as training alone) [27, 28]. It is therefore necessary to shift the efforts of health educators, from traditional training methods to practical ways of increasing self-efficacy.

The results of study indicated that the educational intervention program was able to increase the positive feelings and reduce the negative feelings in the experimental group. The findings of Low et al. suggest that creating a positive emotion in the patient due to receiving support from health care providers creates a sense of security, which ultimately enables the patient to easily accept recommendations regarding treatment options [29]. In addition, health care staff, by using positive emotions, can empower both patients and their families with the knowledge they need to deal with the management of the current status of the disease.

An appropriate strategy for enhancing the positive emotions and reducing the negative ones in patients is to communicate with those who have already been treated using the same therapeutic process and who are satisfied with their HCSB [30]. Besides that, educational interventions in which multiple educational methods and interactive discussions are used can better combat negative emotions and inappropriate perceptions about the treatment process [31].

The findings of this study showed that educational intervention increased the mean score of the interpersonal influences construct in women in experimental group compared to control group. A large number of studies have shown the association between interpersonal influences and their direct impact on health and receiving health care [16, 32]. Supporting relationships on the part of the immediate family members, in particular the spouse, have a positive impact on seeking treatment. Thorburn et al. suggests that, even if the husband of a woman does not support her, other women of the immediate family or her friends can be a strong source of support, and an appropriate pattern of sharing the appropriate information for referring to the health care system [33], which is similar to the strategy used in our study.

The mean score of situational influences in experimental group was significantly different from the pre-intervention stage, but this increase was not statistically significant in comparison with control group. It may be due to the role of some social factors which should be considered in addition to environmental factors. Ensor et al. stated even with the presence of health services and appropriate geographical access, people may not use them, because the health services are not in accordance with their culture and needs [34]. Temmerman et al. also argues that legal and political constraints can limit women’s access to health services [35]. These limitations are more substantial in developing countries, and also promote current gender discriminations in the community. Therefore, community-based, long-term interventions are needed for a longer-term and wider level to increase social empowerment associated with one’s behavior.

After intervention, the mean scores of behavioral preferences and competitive demands significantly decreased in the women in experimental group compared to control group. Jayapalan et al. suggest that education and information in health centers and in the community should be increased to reduce the use of alternative treatments. The public health sector should offer affordable and high quality services. Health planners and managers should be aware of the concerns that patients have on healthcare issues such as access to medicines and confidentiality, that make them evade referring to the health care system and instead seek out alternative treatments [36].

The results of study showed educational intervention, by increasing the women’s ability to manage daily life priorities and increasing control over unforeseen events, reduced the score of immediate competing demands and thus helped improve their decisions in challenging situations. Blanc observed that empowering women had a profound effect on the use of health services and could improve women’s reproductive health [37]. It can be therefore argued that in general, strategies that increase individual self-efficacy to resolve barriers can also partly contribute to reducing immediate competing demands. Regarding the commitment to plan, conducting educational intervention helped increase women’s intention to set goal to start and maintain behavior. When health professionals seek to create behavior changes in the target group, the goal setting is usually comprising one part of their health promotion interventions. Similar to the findings of the present study, the study of Rutter et al. showed that a useful approach to achieving desirable behavior is a process in which individuals divide the behavior in question into smaller goals. This will increase the intent of reaching the ultimate goal [38]. In fact, when women decide to use primary care services, important barriers most often reduce their attending the health care system. Planning for setting goals is the same as the approach that seeks to help women take into account the ways of overcoming such problems in certain steps, and to strengthen their intent to use health care.

Finally, after intervention, the mean score of HCSB in experimental group increased compared to control group, indicating that the designed intervention was appropriate for the behavior. In fact, the HCSB is improved when a person decides to seek health care services in the formal health service resources immediately after observing illness symptoms and interpreting them appropriately. But this behavior is not a mere decision-making process, but rather, in order to achieve this purpose, in addition to behavioral characteristics, other factors that can entail family members’ behaviors, and norms and expectations of the community as well as the characteristics and behaviors of the health service provider should also be taken into account [39]. Therefore, when our goal is to improve a health promoting behavior, we need to comprehensively and holistically examine all the factors affecting the behavior to achieve success.

In the present study, there was a significant difference between the mean scores of referring to the health care system after observing some medical symptoms related to mental disorders, urogenital problems, heart diseases, or some non-specific symptoms before and after the intervention in the experimental group. There is evidence, show that using health intervention strategies such as consultation and education, sharing key messages and mass media campaigns causes a significant increase in appropriate seeking medical care [40–42]. Educational programs with the aim of raising awareness about the warning symptoms and signs of diseases, that may not seem serious to people is a very cost-effective approach for governments and countries. The important point for the successful implementation of these interventions is that these programs should be culturally sensitive and accessible to all individuals.

### Strengths and limitations

One of the strengths of the present study was the use of women’s participation throughout all stages of the intervention (including developing the questionnaire; choosing the appropriate educational strategies and materials that tailored to suit the needs and preferences of them through the needs assessment; implementation; and evaluation). Another strength of this study is the use of a researcher-made questionnaire with a theoretical framework based on HPM which is a comprehensive model for health-related behaviors.

One limitation of the study is the use of a self-report tool and therefore the respondents’ responses may have been subject to their personal interpretations. To mitigate this problem, the tool has been continuously reviewed by a panel of experts in order to develop a comprehensive questionnaire. In addition, participants in this study were selected from women of reproductive age in urban areas, and it is therefore recommended to replicate the study among women in the rural or other cultural settings after modification of educational interventions to suit illiterate women.

## Conclusion

The results of our study show that strategies and educational programs based on the HPM have a positive impact on HCSB and its associated factors. The intervention will be successfully performed when the constituents of the intervention are culturally sensitive and it is accessible to all people in the community. With increasing women’s involvement in implementing interventions, likelihood of conducting the desired behavior also increases. Using such a framework by researchers and, in particular, health education program implementers can help not only improve this behavior in different societies, but also contribute to the success of other health promotion programs.

## Data Availability

The data sets used and/or analyzed during the current study are not publicly available due to confidentiality of data and subsequent research, but are available from the corresponding author on reasonable request.

## Abbreviations

HSCB: Health Care Seeking Behavior
HPM: Health Promotion Model
CVR: Content validity rate
CVI: content validity index
